# Breaking Grounds: A Comprehensive Analysis of Cutting-Edge Treatments for Primary Biliary Cirrhosis/Primary Biliary Cholangitis With Futuristic Treatments

**DOI:** 10.7759/cureus.79582

**Published:** 2025-02-24

**Authors:** Asad Ali Khan, Furqan Ul Haq, Qazi Muhammad Farooq Wahab, Taimur Aslam, Azeem Khalid, Asad Ali

**Affiliations:** 1 Cardiology, Good Hope Hospital, University Hospitals Birmingham NHS Foundation Trust, Birmingham, GBR; 2 Radiation Oncology, Shifa International Hospitals Limited, Islamabad, PAK; 3 Internal Medicine, Hayatabad Medical Complex, Peshawar, PAK; 4 Internal Medicine, Staten Island University Hospital, New York City, USA; 5 Internal Medicine, Aiken Regional Medical Centers, Aiken, USA; 6 Division of Gastroenterology and Hepatology, The State University of New York Upstate Medical University Hospital, Syracuse, USA

**Keywords:** autoimmune gastrointestinal diseases, hepatic and post-hepatic jaundice, liver diseases, primary biliary cholangitis (pbc), primary biliary cirrhosis (pbc)

## Abstract

Primary biliary cholangitis (PBC) is an autoimmune disorder characterized by biliary destruction leading to intrahepatic biliary cholestasis. It predominantly affects women during the fifth and sixth decades. Treatment options have progressed from ursodeoxycholic acid (UDCA) and obeticholic acid (OCA) to liver and stem cell transplant. The objectives include summarizing established and new diagnostic approaches for PBC along with reviewing efficacy treatments, their side effects, and future directions. The treatment of PBC is based on risk stratification, including assessment of the patient’s age, sex, clinical pattern, biochemical and antibody profile, histology, and markers of fibrosis. UDCA and OCA are Food and Drug Administration (FDA) approved first-line and second-line agents. Elafibranor, a recently FDA-approved agent based on its efficacy, was shown in the ELATIVE trial. Seladelpar, currently under FDA review in the ENHANCE III trial, is also used in PBC. Fibrates, a third-line treatment, are found efficacious in different trials. Other treatment options are in phase II/III clinical trials. The question of whether we use immunotherapy has been answered in the NCT02376335 and NCT00746486 trials, stating that rituximab and budesonide cannot be used as no clinical significance is observed. The emergence of new therapies and the potential of combination treatments offer hope for improving outcomes for all patients with PBC. Personalized treatment strategies, continuous monitoring, and a comprehensive approach to symptom management are key to optimizing care and enhancing the quality of life for individuals affected by this chronic liver disease.

## Introduction and background

Primary biliary cholangitis (PBC), previously called primary biliary cirrhosis, is a rare autoimmune chronic hepatobiliary disorder in which there is progressive inflammatory biliary epithelial cell (BEC) destruction, cholestasis, cirrhosis, and ultimately liver failure [1]. Although it can affect anyone of any age or gender, the condition is more prevalent in women and those over 50 years. It affects people of all races, ethnicities, and countries worldwide, and its incidence and prevalence are rising, particularly among women [2,3].

Both genetic and environmental factors are responsible for the pathogenesis of PBC. In PBC, a disease-specific autoantibody called anti-mitochondrial antibody (AMA) targets the cellular mitochondria membranes. This anti-mitochondrial response is primarily responsible for disease pathogenesis, although some non-autoimmune mechanisms may also play a role [4]. Smoking, infections, hormonal therapy, and nail polishing are usually the environmental culprits for the disease pathophysiology. More than 60% of patients are asymptomatic and are diagnosed accidentally when they are found to have abnormal labs, i.e., elevated alkaline phosphatase (ALP), positive AMA, or cholestatic liver pattern labs, by chance [5]. Those who are typically symptomatic manifest with signs and symptoms of chronic cholestasis, i.e., fatigue, pruritus, jaundice, xanthomas, xanthelasmas, and asthenia. Diagnosis of PBC is established with a detailed history, physical examination, lab tests [anti-nuclear antibody (ANA), AMA, ALP, and gamma-glutamyl (GGT)], imaging tests [magnetic resonance cholangiopancreatography (MRCP) and ultrasound (US)], and liver biopsy [6].

A liver biopsy is not required for PBC diagnosis, and diagnosis can be established without a liver biopsy if other criteria are met. Also, AMA positivity alone is not sufficient to make the diagnosis of PBC. Complications of PBC include hepatocellular complications and systemic complications [7]. Hepatocellular complications include cirrhosis, portal hypertension, and hepatocellular carcinoma. Systemic complications include cognitive dysfunction, sicca syndrome, arrhythmia and ventricular dysfunction, nephropathy, osteoporosis, fractures, and increased risk of malignancies [8].

## Review

Methods

This review concentrates on treatment options and breakthroughs in the diagnosis of PBC. Using precise keywords and MeSH phrases linked to PBC diagnosis and therapies, a thorough search was conducted throughout PubMed, Cochrane Library, EMBASE, and ClinicalTrials.gov. Cohort studies, phase II/III clinical trials, and randomized controlled trials were among the studies that qualified for inclusion, while case reports and animal studies were excluded. Study design, patient demographics, diagnostic techniques, treatments (first-line, second-line, and novel agents), and clinical outcomes were all covered by the data extraction process.

Epidemiology

The disease is more common among females, with a female-to-male ratio ranging from 4 to 5:1 and 9 to 10:1, and those above 50 years, but it can occur across any age and gender group [9]. The incidence and prevalence of PBC are on the rise, especially among the female patient population. The average incidence rate is 3.0 cases per 100,000 individuals per year, with a prevalence rate of 21.05 cases per 100,000 individuals. PBC affects individuals of all ethnicities, races, nationalities, and distribution worldwide [10].

Etiology and pathophysiology of PBC

The exact etiology and mechanism by which PBC develops remain unclear. PBC is a multifactorial disease, and both genetic and environmental factors are involved in the disease pathophysiology. In PBC, the primary target of damage is to the BECs. This has been associated with the immunobiology of BECs in persons who are genetically predisposed and subjected to environmental stressors. BECs overexpress microRNA-506 (miR-506), resulting in inadequate secretion of biliary bicarbonate secretion that manifests as cholestasis. In addition, it targets exclusively the messenger ribonucleic acid (mRNA) of anion exchanger 2 (AE2), resulting in cholangiocytes developing PBC-like characteristics. The E2 component of the pyruvate dehydrogenase complex (PDC-E2) is overexpressed and exhibits odd localization as a result of toxic bile acid’s (BA) unregulated infiltration of BECs and promotion of apoptosis. Due to the absence of glutathionylation in apoptotic bodies, PDC-E2 is unaltered during cholangiocyte apoptosis. Circulating AMA would identify the PDC-E2 found in the apoptotic bodies, and the immune complex would then activate the innate immune system in an individual with a genetic predisposition.

Clinical presentation and diagnosis of PBC

**Figure 1:**
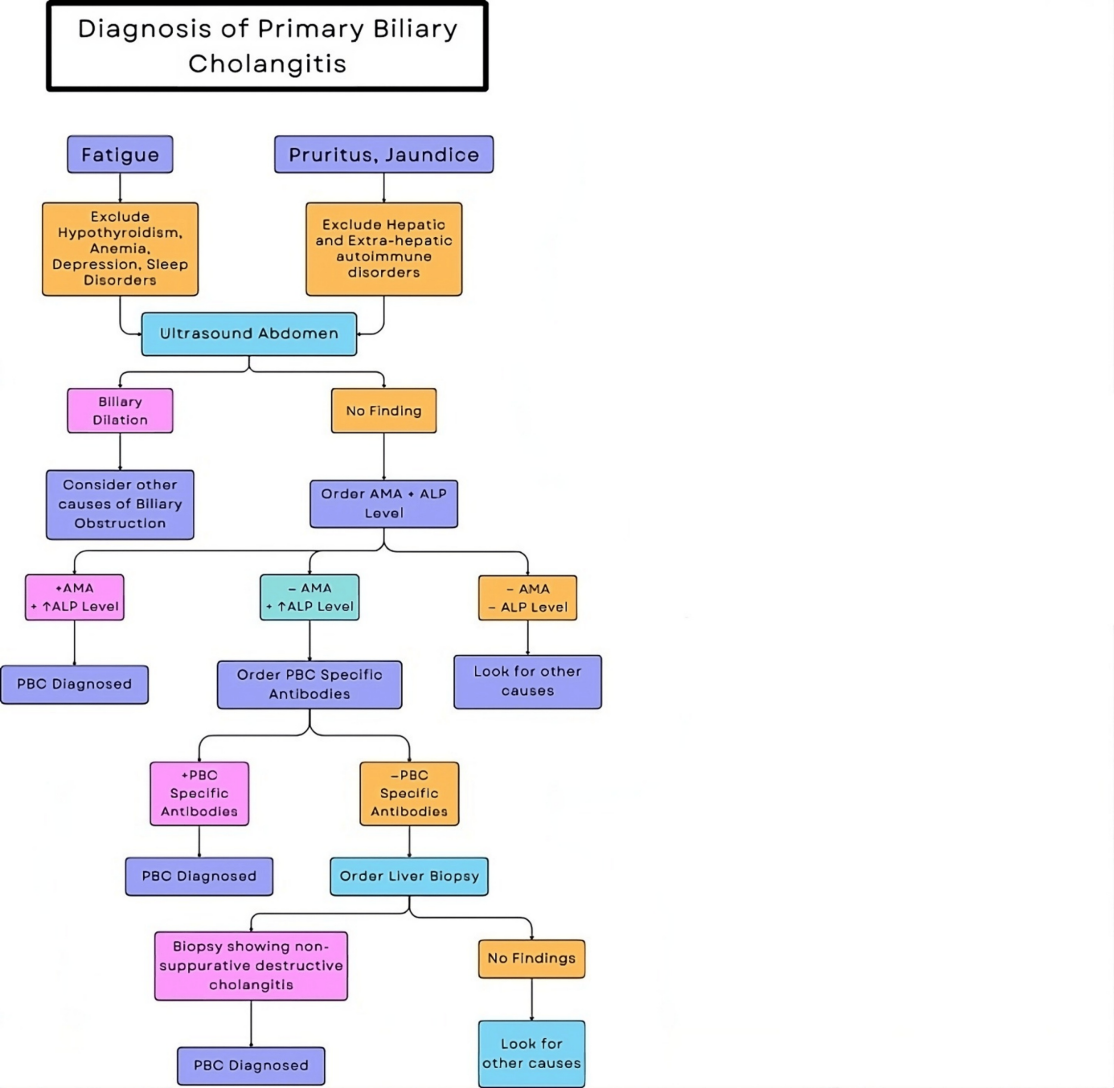
Algorithm for the diagnosis of PBC ALP, alkaline phosphatase; PBC, primary biliary cholangitis; AMA, anti-mitochondrial antibody

Most of the patients with PBC are asymptomatic at the time of diagnosis. Symptomatic patients present with pruritic and fatigue, which are present in up to 70% of patients. Thyroid disorders, anemia, depression, adrenal disorders, and sleep disorders could also cause fatigue and should be considered before attributing symptoms to PBC [4]. Sjogren syndrome, thyroid disorders, celiac disease, and systemic sclerosis are the common autoimmune disorders commonly seen in patients with PBC [11]. In all, 90 to 95% of patients with PBC have positive serology for AMA. Because of its high specificity, PBC diagnosis can be easily made in a patient presented with positive AMA serology and cholestasis without ordering a liver biopsy [12]. Figure 1 presents the algorithmic approach for the diagnosis.

PBC-specific anti-nuclear antibodies like anti-sp100 and anti-gp210 can be found in 50% of AMA-negative patients. This helps in the diagnosing of PBC in AMA-negative individuals [13]. Anti-kelch-like 12 (KLHL12) and anti-hexokinase 1 (HK1) are other PBC-specific antibodies that can be found in PBC patients, but their practical use needs further studies and understanding. The nature of disease and clinical outcomes is exhibited similarly by both AMA-positive and AMA-negative PBC patients [14]. The risk of fat-soluble vitamin deficiencies and lipid disorders is high in PBC patients. Unless there is the absence of serological markers or high suspicion of nonalcoholic steatohepatitis or autoimmune hepatitis, a liver biopsy is not needed for the diagnosis of PBC [15]. The diagnosis of PBC is supported by the absence of other liver and systemic pathologies and chronically high ALP levels but positive serology for AMA. It is also supported by the absence of AMA but positive PBC-specific antibodies in patients with chronically high ALP levels and liver biopsy showing evidence of PBC in a patient with elevated ALP levels without any serological markers H.

Risk stratification and survival analysis

The progression of PBC varies throughout the population; while some see a gradual decline in their condition, others experience severe fibrosis and liver cirrhosis in a matter of years. To determine a patient’s prognosis, it is necessary to evaluate their unique risk of progression. In order to do this, a number of laboratory, serological, clinical, and demographic factors are assessed. Additionally, the disease stage is determined depending on the degree of fibrosis and the patient’s reaction to treatment. Table 1 summarizes the risk stratification [16,17].

**Table 1:** Risk Stratification ACA, anti-centromere antibodies; AIH/PBC OS, autoimmune hepatitis/primary biliary overlap syndrome; AMA, anti-mitochondrial antibodies; APRI, AST-to-platelet ratio index; ELF score, enhanced liver fibrosis score; HK1, hexokinase-1; KLHL12, kelch-like 12 protein; LSM, liver stiffness measurement; MRE, magnetic resonance elastography; ULN, upper limit of normal

Serial no.	Factors	Low risk	High risk
1	Age	˃55 years	˂55 years
2	Sex	Female	Male
3	Clinical pattern	No symptoms	Symptomatic disease
			AIH/PBC OS
			Premature ductopenic varient
4	Antibody profile	AMA	Anti-gp210+
			ACA+
			Anti-HK1+
			Anti-KLHL12+
5	Biochemical panel	Normal bilirubin	↑ Bilirubin
		ALP ˂ 2× ULN	ALP ≥ 2× ULN
			APRI score ˃ 0.54
6	Histology	No/mild fibrosis	Advanced fibrosis/cirrhosis
			Interface hepatitis
			Ductopenia at the diagnosis
7	Non-invasive markers of fibrosis	LSM < 8 kPa/↑ < 2.1 kPa/y	LSM > 15 kPa/↑ > 2.1 kPa/y
		ELF score < 10.0	ELF score ≥ 10.0
		MRE < 4.6 kPa	MRE > 4.6 kPa

Hardie et al. identified PBC patients as low-risk if they have a positive outcome in terms of improvement and as high-risk if they have a poor outcome in terms of PBC development [18]. It is reported that genes associated with apoptosis and cell cytotoxicity are up-regulated, while genes related to complement pathways are down-regulated in high-risk disease patients as compared to low-risk patients. In high-risk patients, the pathways related to T-cell activation, leukocyte migration, apoptosis, and interferon-gamma response were significant. The gene product of CDKN1a, the senescence marker p21WAD1/Cip, shows higher expression levels on the bile ducts in high compared to low-risk patients [18].

Tian et al. carried out a study to explore novel biomarkers of risk stratification for PBC patients utilizing the gene expression omnibus (GEO) database. They identified 166 differentially expressed genes (DEGs), and 15 of them were associated with disease progression (seven up-regulated genes and eight down-regulated genes). Four core-risk-related genes, including TXNIP, CD44, ENTPD1, and PDGFRB, were defined, and three of these genes (TXNIP, CD44, and ENTPD1) showed upregulation on qRT-PCR resulting in the development of a three-gene panel for screening high-risk PBC patients [19].

Treatment of PBC

The treatment of PBC is based on risk stratification, which includes assessment of the patient’s age, sex, clinical pattern, biochemical profile, histology, antibody profile, and markers of fibrosis; based on these, the patients are divided into low-risk and high-risk for PBC [20].

**Table 2:** Treatment of PBC LTF, liver transplantation free; FDA, Food and Drug Administration; PPAR, peroxisome proliferator-activated receptors; ALP, alkaline phosphatase; GGT, gamma-glutamyl transferase; HCC, hepatocellular carcinoma; FGF, fibroblast growth factor; ASBT, apical sodium-dependent bile acid transporter; TGR, Takeda G protein-coupled receptor 5; PDC-E2, pyruvate dehydrogenase complex E2 component

Characteristics	Drug	Mechanism	Effects	Clinical status	Dose	Adverse effects
FDA-approved drugs	Ursodeoxycholic acid (approved in 1997)	Acts in the liver through multiple interrelated pathways	Promotes bile acid excretion, decreases biliary cholesterol, reduces liver damage by bile acid, and improves overall and LTF survival	FDA-approved first-line agent	13-15 mg/kg/day	Nausea, vomiting, diarrhea, flatulence, and sleep problems
	Obeticholic acid (approved in 2016)	Farnesoid X receptor agonist	Inhibits bile acid synthesis, improves enterohepatic circulation, and has anti-inflammatory and anti-fibrotic effects	FDA-approved second-line agent	5-10 mg/day	Pruritus, hepatic decompensation, and lipid derangements
	Elafibranor (approved in 2024)	PPARα and δ agonist	Reduction in the ALP, triglycerides, and cholesterol levels	Recently approved by the FDA based on the ELATIVE trial results	80 mg/day	Headache, nausea, fatigue, diarrhea, and creatinine elevation
Potential future therapies	Seladelpar (MBX-8025)	PPARδ agonist	Improvement in ALP and pruritis	Under FDA review for approval in the ENHANCE phase III trial	10 mg/day	Nil reported
	Bezafibrate	pan-PPAR agonist	Improvement in liver biochemistry, fibrosis, and stiffness and alleviates pruritus	Third-line treatment in the BEZURSO trial adds on therapy with UCA	Trial with 400 mg/day	Myalgia, increased creatinine, and hepatotoxicity
	Fenofibrate	PPARα agonist	Improves liver biochemistry, pruritus, and LTF survival and reduces fibrosis	ChiCTR1800020160 trial	Tested dose is 200 mg/day	NA
	Pemafibrate	PPARα agonist	Improvement in ALP and GGT	NCT06247735 is an ongoing phase 2 trial	NA	NA
	Tropifexor (LJN452)	Farnesoid X receptor agonist	Prevents bile acid-mediated liver damage and fibrosis	Under clinical trials and NCT02516605 is a notable phase II trial	30-90 ug daily doses have been tested	Pruritus
	Cilofexor (GS-9674)	Farnesoid X receptor agonist	Reduction in ALP levels observed	Phase II clinical trial (NCT02943447)	30 and 100 mg doses have been tested	Pruritis
	Aldafermin (NGM282)	FGF19 analogue	Improvement in ALP, transaminase, and liver damage and protection from fibrosis and HCC progression	NCT02026401 is a phase II trial	0.3 and 3 mg dose groups	NA
	Saroglitazar	PPARα and γ agonists	Improvement in ALP and cholestatic markers	NCT05133336 is an ongoing phase IIb/III study	1 and 2 mg doses are being tested	Elevation in aminotransferases
	Linerixibat (GSK2330672)	ASBT inhibitor	Reduces bile acid and improves pruritus	GLISTEN is an ongoing phase III trial (NCT04950127)	NA	NA
	Volixibat	ASBT inhibitor	-	NCT05050136 is an ongoing phase II trial	NA	NA
	Setanaxib (GKT137831)	Inhibits NOX1 and 4	Improves cholestasis, liver fibrosis, and fatigue	NCT05014672 and NCT03226067 are phase II trials	Doses of 400-1600 mg have been tested	NA
Ineffective therapies	Rituximab	Anti-CD20 mab	Improves ALP levels but no clinically significant effects seen	NCT02376335 was a phase II trial, which is now closed	1000 mg tested	NA
	Budesonide	Immunosuppressant	Improvement in cholestatic markers	NCT00746486 was a phase III trial, which is now terminated due to underpower	Tested dose is 9 mg/day	Arthralgia, hypertension, muscle spasms, weight gain, peripheral edema, and dyspepsia
Preclinical agents	BAR501INT-777 and INT-767	TGR5 agonists	Reduces inflammation	-	-	-
	5-Aza-2-deoxycytidine (DAC)	Maintains balance of Treg/Th17	Anti-inflammatory and promotes tolerance	-	-	-
	CNP-104 nanoparticle	Targets PDC-E2	-	-	-	-
	Simtuzumab	Monoclonal antibody against LOXL2	Improves liver fibrosis	-	-	-

For the past few decades, different regimes have been used for PBC. Ursodeoxycholic acid (UDCA) is a Food and Drug Administration (FDA) approved first-line agent acting on the promotion of BA excretion and decreases cholesterol, which reduces biliary damage with a dose of 13-15 mg/kg/day, slightly risk of nausea and vomiting though flatulence and diarrhea are common [20]. Obeticholic acid (OCA) is an FDA-approved second-line agent with a dose of 5-10 mg/day, though it causes pruritus and lipid derangement. The efficacy of Elafibranor, a recently FDA-approved agent, is shown in ELATIVE trial results with a dose of 80 mg/day, which causes headache, diarrhea, and raised creatinine. Seladelpar, which is under review for FDA approval in the ENHANCE III trial, is also used in PBC at a dose of 10 mg/day. Fibrate, a third-line treatment like bezafibrate, fenofibrate, or pemafibrate, was tried and found efficacious in different trials, such as the BEZURSO trial, ChiCTR1800020160 trial, and NCT06247735 that is an ongoing phase II trial.

Tropifexor is in phase II clinical trial, is under review, and is used in a daily dose of 30-90 ug, which causes pruritus [21]. Cilofexor is also in phase II clinical trial and is used in a dose of 30-100 mg, which also causes pruritus. Linerixibat, an apical sodium-dependent BA transporter (ASBT) inhibitor, and volixibat, an ASBT inhibitor, are under phase III and II trials, respectively. Setanaxib, which is a NOX 1 and 4 inhibitor, is also under phase II trial [22].

The question of whether we use immunotherapy has been answered in the NCT02376335 and NCT00746486 trials, stating that we cannot use rituximab and budesonide as no clinical significance is seen, along with under power study. Other immunosuppressive and monoclonal antibodies, such as BAR501INT-777 and INT-767, 5-aza-2-deoxycytidine, and CNP-104 nanoparticles, are under study in the treatment of PBC, and their results are awaited [23].

Discussions

PBC is an autoimmune disease, though the treatment is based on improving liver functions and slowing down the progression of worsening liver function tests. The treatment landscape has significantly evolved over the recent decades, reflecting advancement in our understanding of the disease pathophysiology and patient needs. This review synthesizes the current evidence on therapeutic options, highlighting both the established and emerging treatments [24].

Most PBC patients respond well to UDCA, as one study has shown improved survival without transplantation in patients treated with UDCA compared to untreated patients [hazard ratio (HR): 0.46; 95%CI, 0.40 to 0.52; p < 0.001). However, some patients develop advanced liver cirrhosis even after receiving treatment. Multiple studies have confirmed its efficacy in improving biochemical markers, slowing disease progression, and enhancing survival rates [25]. The dosage is kept between 13 and 15 mg/kg and can be given as a single dose orally. The majority of response criteria take into account both bilirubin and ALP, which are useful in clinical practice (higher ALP readings may be utilized as a risk marker of early but quickly progressing PBC, whereas bilirubin tends to be an indicator of advanced disease). UDCA is the first-line approved drug, though it causes nausea, vomiting, flatulence, and diarrhea. The need for second-line treatment has been recommended after the use of UDCA [22].

OCA has emerged as the first approved second-line therapy in patients with inadequate response to UDCA. Clinical trials have demonstrated its efficacy in further reducing ALP levels and other markers of cholestasis. OCA acts by activating the farnesoid X receptor (FXR), which regulates BA synthesis and transport. However, concerns about pruritus and potential long-term cardiovascular risks necessitate careful patient selection and monitoring. This review highlights the importance of personalized therapy, considering both the benefits and potential side effects of OCA [26].

Fibrates, particularly fenofibrate and bezafibrate, have promise in PBC treatment, especially in combination with UDCA. These agents, which activate peroxisome proliferator-activated receptors (PPARs), have demonstrated improvements in liver biochemistry and symptoms. However, their impact on long-term outcomes, such as liver transplantation rates and overall survival, requires further investigation. Ongoing studies and real-world evidence are essential to establish their role in the standard treatment algorithm [27].

In emerging therapies, there are several novel therapeutic agents currently under investigation, targeting various pathways involved in PBC pathogenesis. Among these, seladelpar, a potent PPAR-delta agonist, has shown efficacy in reducing ALP, marked improvement in liver biochemistry, and improved liver histology with a favorable safety profile [28]. Another novel agent is norUDCA, which is a modified BA with anti-inflammatory and anti-fibrotic properties and is currently in clinical trials with the dose maintained at 1500 mg, resulting in a significant reduction of ALT within 12 weeks of treatment as compared to placebo.

Liver-Targeted Immunotherapies

Liver-targeted immunotherapies are designed to modulate the immune response specific to PBC, offering the potential for disease modification. The review discusses the potential of these emerging therapies to address unmet needs in PBC treatment, particularly for patients who do not respond adequately to existing therapies. Clinical trials of pipeline medications and animal research have shown encouraging results in halting the course of illness. In the early stage of the disease, treatment is targeted toward immune-mediated pathogenesis and anti-inflammatory therapies. On the other hand, in the late stage of the disease, defined by fibrosis and cirrhosis, anti-cholestatic and anti-fibrotic therapies are utilized [29].

In combination therapy for PBC, given the heterogeneity in treatment response, combination therapy represents a promising approach. Combining agents with complementary mechanisms of action could enhance therapeutic efficacy and mitigate adverse effects. Preliminary studies on combinations, such as UDCA with fibrates or OCA, have shown encouraging results, but larger, long-term studies are needed to confirm these findings [30].

Future directions

Future research should focus on understanding the predictors of treatment response, optimizing combination therapies, and developing biomarkers for early diagnosis and treatment monitoring. Moreover, the integration of patient-reported outcomes in clinical trials will provide insights into the impact of therapies on quality of life. Collaborative efforts in multicenter trials and real-world studies are essential to advance the treatment paradigm for PBC.

## Conclusions

This review highlights the significant progress made in the treatment of PBC while acknowledging the challenges that remain. The emergence of new therapies and the potential of combination treatments offer hope for improving outcomes for all patients with PBC. Personalized treatment strategies, continuous monitoring, and a comprehensive approach to symptom management are key to optimizing care and enhancing the quality of life for individuals affected by this chronic liver disease.
